# The common European mosquitoes *Culex pipiens* and *Aedes albopictus* are unable to transmit SARS-CoV-2 after a natural-mimicking challenge with infected blood

**DOI:** 10.1186/s13071-021-04578-9

**Published:** 2021-01-22

**Authors:** Claudia Fortuna, Fabrizio Montarsi, Francesco Severini, Giulia Marsili, Luciano Toma, Antonello Amendola, Michela Bertola, Alice Michelutti, Silvia Ravagnan, Gioia Capelli, Giovanni Rezza, Marco Di Luca, the Working Group

**Affiliations:** 1 Department of Infectious Diseases, Istituto Superiore di Sanità, Rome, Italy; 2 Istituto Zooprofilattico Sperimentale delle Venezie, Legnaro, PD Italy; 3 Ministry of Health, Rome, Italy

**Keywords:** SARS-CoV-2, *Aedes albopictus*, *Culex pipiens*, Vector competence, Mechanical transmission

## Abstract

**Background:**

On 11 March 2020, the World Health Organisation (WHO) declared the coronavirus disease 2019 (COVID-19) outbreak to be a pandemic. As the mosquito season progressed, the understandable concern that mosquitoes could transmit the virus began to increase among the general public and public health organisations. We have investigated the vector competence of *Culex pipiens* and *Aedes albopictus*, the two most common species of vector mosquitoes in Europe, for severe acute respiratory syndrome coronavirus 2 (SARS-CoV-2). Due to the very unusual feeding behaviour of *Ae. albopictus*, we also evaluated the role of this mosquito in a potential mechanical transmission of the virus.

**Methods:**

For the vector competence study, mosquitoes were allowed to take several infectious blood meals. The mosquitoes were then collected and analysed at 0, 3, 7 and 10 days post-feeding. For the mechanical transmission test, *Ae. albopictus* females were allowed to feed for a short time on a feeder containing infectious blood and then on a feeder containing virus-free blood. Both mosquitoes and blood were tested for viral presence.

**Results:**

*Culex pipiens* and *Ae. albopictus* were found not be competent vectors for SARS-CoV-2, and *Ae. albopictus* was unable to mechanically transmit the virus.

**Conclusions:**

This is the first study to show that the most common species of vector mosquitoes in Europe do not transmit SARS-CoV-2 and that *Ae. albopictus* is unable to mechanically transmit the virus from a positive host to a healthy host through host-feeding.
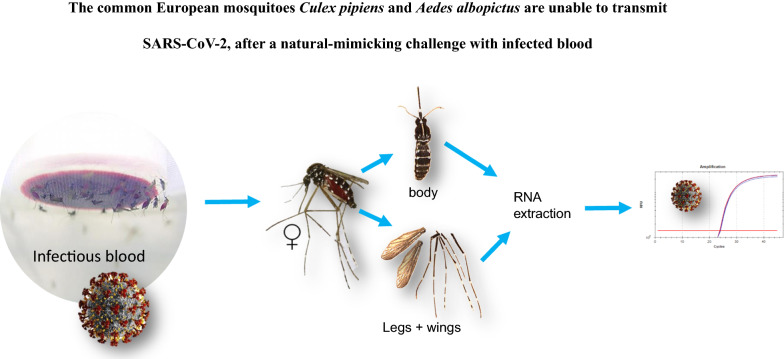

## Background

On 31 December 2019, Chinese health authorities reported a cluster of severe pneumonia cases of unknown etiology in the city of Wuhan (Hubei province, China). On 9 January 2020, an agent which is phylogenetically grouped in the severe acute respiratory syndrome coronavirus (SARS-CoV) clade was reported to be the causative agent of this outbreak (SARS-CoV-2). The disease associated with the virus was considered to be novel coronavirus disease and referred to as coronavirus 2019 (COVID-19). On 11 March 2020, the World Health Organisation (WHO) declared COVID-19 a pandemic [1].

SARS-CoV-2 replicates primarily in the respiratory tract of infected patients, but also in a variety of other cell types albeit with less efficiency. In addition, in some cases, especially in those with severe disease, the virus has been detected in the peripheral blood [2–4], raising concern about the risk of vector-borne transmission. In this context, several previously published studies had demonstrated the possible role of blood-sucking arthropods, including mosquitoes, in the transmission of viruses other than arboviruses for which they are the known vectors [5–7]. However, the possibility that mosquitoes can transmit SARS-CoV-2 was declared unlikely by the WHO right at the beginning of the epidemic [8], an assessment that was subsequently confirmed in experimental studies which showed that SARS-CoV-2 is unable to replicate in *Aedes* mosquito cells* in vitro* [9] or in intrathoracically infected *Aedes aegypti*,* Ae. albopictus* and *Culex quinquefasciatus* in USA [10]. To date, no data are available on the vector competence of *Culex pipiens* for SARS-CoV-2. This species is one of the most common human-biting mosquitoes in Europe, where it is responsible for the transmission of the West Nile and Usutu viruses [11]. In addition, a possible role of mosquitoes as mechanical vectors of viral etiological agents, including respiratory viruses, has already been observed [12, 13]. Indeed, the propensity of some mosquito species, such as *Ae. albopictus*, to take several meals on different hosts, even over a short period of time, can increase the risk of infectious disease transmission by increasing the frequency of contact with hosts [14]. In fact, this diurnal species does not make a complete meal with a single bite but, if disturbed, it can make short and frequent meals on the same or different hosts.

Based on these known characteristics, we have assessed the vector competence of the two most common mosquito species in Europe, *Cx. pipiens* and *Ae. albopictus*, for SARS-CoV-2 after oral infection. We also evaluated potential mechanical transmission of the virus through *Ae. albopictus*. The study was carried out by the Istituto Superiore di Sanità (ISS) and the Istituto Zooprofilattico Sperimentale delle Venezie (IZSVe).

## Methods

For the vector competence analysis, *Ae. albopictus* and *Cx. pipiens* colonies were fed on an infectious blood meal using an artificial membrane feeding system [15]. SARS-CoV-2 used for the experiments was isolated from patients infected in Italy during the COVID-19 pandemic. These patients had been hospitalised with an acute respiratory illness (pneumonia); all had shown bilateral lung involvement with ground-glass opacity and required intensive care. Biological samples from these patients had been confirmed to be SARS-CoV-2 positive by the National Reference Laboratory (NRL) of the ISS [16].

 The infectious blood meal was prepared by ISS. For these experiments, the virus was diluted 1:3 in rabbit blood to a final virus concentration of 1.20 × 10^6^ plaque-forming units (PFU)/ml. Although the SARS-CoV-2 titer detected in the blood of these patients was generally lower than the concentration used in the study, an artificial membrane feeding system requires a higher viral titer due to the inevitable yield limits of experimental systems [15]. The mosquitoes used in the experimental infections were from long-established laboratory colonies of *Cx. pipiens* and *Ae. albopictus* maintained at the ISS Insectary. Mosquitoes aged 5–8 days were selected, and cages containing 80 females of each species were set up. To stimulate blood-sucking behaviour, the mosquitoes were starved for 12 h before the experimental infection by depriving them of the sucrose solution which they normally fed on. The infection experiment was performed in a Biosafety Level 3 Laboratory (BSL3) cabinet at 28 °C and a relative humidity of about 70%. Female mosquitoes were allowed to feed for 120 min through a pig intestine membrane covering a glass feeder containing the blood that was maintained at 37°C by a warm water circulation system. During the experiment two cages of *Cx. pipiens* and *Ae. albopictus* were allowed in parallel to take an uninfected blood meal; these mosquitoes were subsequently monitored during the study, as controls, to verify the survival of mosquito populations under experimental conditions.

 Experiments at IZSVe were performed on *Ae. albopictus* and followed the same protocol adopted by ISS with slight modifications. In these experiments, the virus was diluted 1:20 to achieve a final concentration of 10^6^ PFU/ml in defibrinated sheep blood and a different artificial feeder device was used (Hemotek Inc., Blackburn, UK) for the blood meal. Two cages of 80 females from long-established laboratory colonies of *Ae. albopictus* from the Insectary of Entostudio Srl (Ponte San Nicolò, Italy) were set up. After the meal only fully engorged females were selected in a glove box and then transferred and maintained in a climate chamber (26 ± 1 °C; 70% relative humidity; 14/10-h light/dark cycle) with a 10% sucrose solution. About 6–12 mosquitoes of both species were individually analysed at each Institute on 0, 3, 7 and 10 days post-infection (dpi). On day 0, specimens of each mosquito species were tested to confirm the ingestion of viral particles; at 3, 7 and 10 dpi, each mosquito was individually examined by separating the body from the legs and wings. Mosquito bodies were investigated to evaluate the infection rate (IR), calculated as the number of SARS-CoV-2 RNA-positive bodies compared to the total number of females tested. Legs and wings were tested to assess the dissemination rate (DR), calculated as the number of samples with SARS-CoV-2 RNA-positive legs and wings (pooled together) among infected mosquitoes.

SARS-CoV-2 RNA load was assessed by quantitative reverse transcription PCR (qRT-PCR). In accordance with the procedure adopted by ISS, viral RNA was extracted using the QIAamp viral RNA Mini Kit (Qiagen, Hilden, Germany). Aliquots (5 µl) of extracted RNA were analysed for the N2 gene by qRT-PCR, using the protocol from the U.S. Centers for Disease Control and Prevention (CDC) [17]. SARS-CoV-2 titer was assessed by crossing point values compared with a standard curve obtained from tenfold serial dilutions of a virus stock of known concentration (3.6 × 10^6^PFU/ml). In accordance with the procedure adopted by IZSVe, viral RNA was extracted using the MagMAX™ Pathogen RNA/DNA Kit (Thermo Fisher Scientific, Waltham, MA, USA) with the automated extraction instrument KingFisher Flex (Thermo Fisher Scientific). Aliquots (5 µl) of extracted RNA were analysed for the E gene by real-time RT-PCR (rRT-PCR) [18]. The samples were exchanged between the two Institutes for cross-analysis to strengthen the molecular analysis and make the results more reliable.

The mechanical transmission test was performed using the same membrane feeding system and under the same conditions as described above. Eighty *Ae. albopictus* females, previously starved, were allowed to feed for a short time, with constant disturbance, on a feeder containing infectious blood at a concentration of 1.2 × 10^6^ PFU/ml. After 2 min, the feeder was removed and replaced immediately afterwards with a feeder containing 3 ml of virus-free blood, and the mosquitoes were allowed to complete the meal until they became replete. After the meal, the blood was collected and analysed for the viral presence. The engorged female mosquitoes were killed and stored at − 80°C for subsequent analysis. The blood was subjected to RNA extraction and amplification by qRT-PCR using both protocols.

## Results

**Figure 1.**
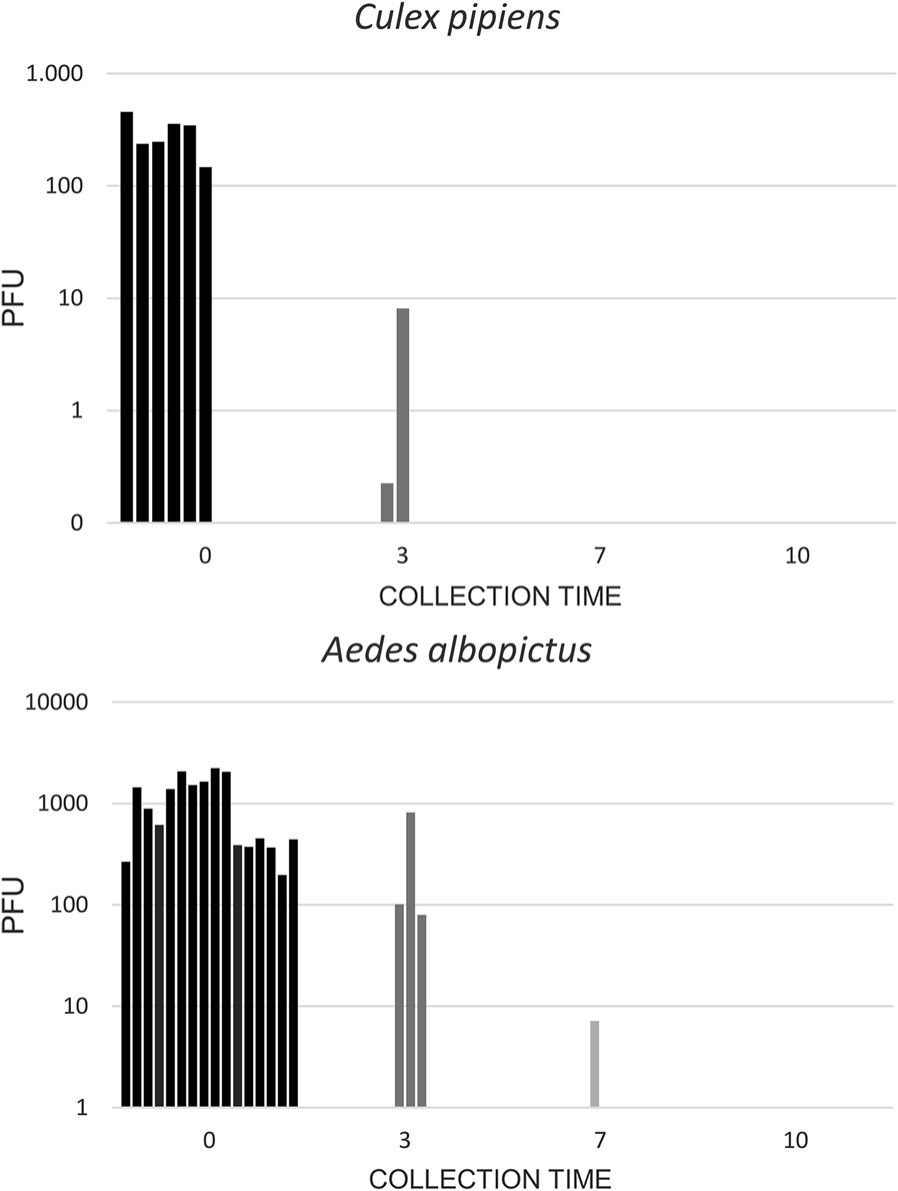
Viral titers in *Aedes albopictus* and *Culex pipiens* bodies analysed at different days post‐infection with severe acute respiratory syndrome coronavirus 2

The vector competence analysis showed that immediately after taking the infectious blood meal (0 dpi), all tested *Ae. albop*ictus and *Cx. pipiens* mosquitoes tested positive in the qRT-PCR analyses, confirming the ingestion of viral particles. Viral titers were variable, especially for *Ae. albopictus*, with a minimum value of 1.97 × 10^2^ PFU equivalents up to a maximum value of 2.23 × 10^3^ PFU equivalents, depending on the blood meal taken by mosquitoes (Fig. 1). At 3 dpi, three of the twenty-two *Ae. albopictus* and two of the ten *Cx. pipiens* analysed were infected, showing an IR value of 13.6 and 20%, respectively (Table1). In *Ae. albopictus* bodies, the viral titers were 7.94 × 10, 1.01 × 10^2^ and 8.18 × 10^2^ PFU equivalents. In the two positive *Cx. pipiens* bodies, the titers were considerably lower, at 8.10 and 0.22 PFU equivalents. At 7 dpi, only one of the 20 *Ae. albopictus* bodies tested positive, with a viral titer of 7.1 PFU equivalents; to the contrary, no viral genome was found in *Cx. pipiens* at this time-point. At the last collection time, 10 dpi, no positive bodies were found in either mosquito species. The virus was never detected in the pooled legs + wings samples of the analysed *Ae. albopictus* and *Cx. pipiens* specimens (Table 1). During the experiment, the potentially infected mosquitoes of both species were allowed to lay eggs (first gonotrophic cycle). Larvae were reared and maintained up to the adulthood following standardised procedures [15]. Two pools of ten larvae per species were processed for the N2 gene by qRT-PCR and for the E gene by rRT-PCR; all larvae tested negative for SARS-CoV-2. Mosquito adults (15 *Ae. albopictus* and 13 *Cx. pipiens*) of both species that emerged from the first gonotrophic cycle were also analysed for viral presence by qRT-PCR; all tests were negative.

**Table 1.** Infection and dissemination rates of *Aedes albopictus* and *Culex pipiens* artificially infected with severe acute respiratory syndrome coronavirus 2

Days post-infection	*Aedes albopictus*		*Culex pipiens*	
	Infection rate (%)^a^	Disemination rate (%)^b^	Infection rate (%)^a^	Disemination rate (%)^b^
0	12/12 (100%)	-	6/6 (100%)	-
3	3/22 (13.6%)	0/22 (0%)	2/10 (20%)	0/10 (0%)
7	1/20 (5%)	0/20 (0%)	0/10 (0%)	0/10 (0%)
10	0/22 (0%)	0/22 (0%)	0/11 (0%)	0/11 (0%)

^a^Number of virus-positive mosquito bodies/number of bodies tested

^b^Number of virus-positive legs + wings/number tested

The results of the mechanical transmission analysis did not highlight the presence of viral genome in the virus-free blood on which the *Ae. albopictus* mosquitoes completed the meal immediately after feeding partially on a feeder containing infectious blood. The engorged mosquitoes were individually analysed by qRT-PCR. We analysed 21 mosquitoes, all SARS-CoV-2 positive. The viral titer detected in individual mosquitoes was variable, ranging from 6.32x10^2^ to 3.44x10^1^ PFU equivalents, depending on ingested viral particles by mosquitoes during the disturbed infectious blood meal.

## Discussion

Human infection with SARS-CoV-2, a novel coronavirus of probable zoonotic origin [19], is characterised by a spectrum of clinical conditions, ranging from mild upper airway respiratory symptoms to severe life-threatening pneumonia. In Italy, in February 2020, with the approach of the favorable season for the development of mosquitoes, the COVID outbreak aroused the concern of the general population and health authorities for a potential transmission of the disease through mosquito bites. This concern appeared to be justified by the detection of SARS-CoV-2 in the blood of some patients, with positivity in human sera and/or whole blood varying from 8 [2] to 40% [20].

Among mosquito species, *Ae. albopictus* and *Cx. pipiens* are widespread in Italy and are efficient vectors for some of the most relevant and well-known arboviruses, such as chikungunya and West Nile viruses. In addition, *Ae. albopictus*, due to its marked aggressiveness and anthropophilia, its broad distribution at very high local densities and its trophic diurnal behavior, could represent a powerful mechanical vector of SARS-CoV-2 in an urban environment, especially in the presence of a high circulation of SARS-CoV-2 among the general population and in absence of mosquito control activities that were suspended during the lockdown. Based on these assumptions, we tested the potential vector competence for SARS-CoV-2 of the most common and widespread mosquito species in Europe, *Ae. albopictus* and *Cx. pipiens*, using a membrane feeding system to simulate a more natural blood meal. The SARS-CoV-2 genome was detected in the bodies of both species at 3 dpi, but at 7 dpi it was detected in the body of only one *Ae. albopictus* specimen, at a very low viral titer. The decreasing trend of the viral titer in the mosquitoes tested at the different collection times shows that the virus is progressively digested. A blood meal is digested by a mosquito on average after 3 days, but in many species this phase can last longer and exceed 5 days [21], as in one of the mosquitoes analysed in our study, in whose body viral RNA was detected on the seventh day after the infectious meal. However, it is important to note that viral RNA was detected at a very low titer in the body of the latter mosquito body, and that it was undetectable in the wings and legs. This result shows that SARS-CoV-2 did not spread to the mosquito haemocele, but remained confined to the midgut, suggesting that the localisation and its decrease could be attributable to the digestion process and activation of the mosquito immune system [22].

As expected, analysis of *Ae. albopictus* and *Cx. pipiens* larvae and adults born from eggs deposited by potentially infected mosquitoes revealed that there was no vertical transmission of the virus.

We also investigated the possible mechanical transmission of SARS-CoV-2 by the *Ae. albopictus*, a mosquito species with a diurnal and peculiar trophic activity which, if disturbed, can take short and frequent meals on the same or different hosts. *Aedes albopictus* females that had partially fed on the infectious blood and which were disturbed but soon afterwards were allowed to complete their meal with virus-free blood were all positive for the viral genome, confirming the ingestion of viral particles by the mosquitoes. To the contrary, virus-free blood was analysed and no viral genome was detected. This result suggests that mosquitoes which are partially engorged with a first infectious blood meal are unable to mechanically release the virus immediately thereafter upon biting an uninfected host.

## Conclusions

In conclusion, our findings provide additional and definitive scientific evidence that SARS-CoV-2 cannot replicate and spread in mosquitoes, as shown in a natural context, i.e. through direct ingestion of an infectious blood meal. This study also shows for the first time that *Ae. albopictus* is unable to mechanically transmit the virus to a healthy host after first feeding on a SARS-CoV-2-positive host, even in the hypothetical case of very high viremia.

## Data Availability

Not applicable

## Abbreviations

BSL3: Biosafety Level 3 Laboratory
CDC: Centers for Disease Control and Prevention
DR: Dissemination rate
IR: Infection rate
ISS: Istituto Superiore di Sanità
IZSVe: Istituto Zooprofilattico Sperimentale delle Venezie
PFU: Plaque-forming units
qRT-PCR: Quantitative reverse transcription PCR
WHO: World Health Organisation

## References

[1] Novel coronavirus disease 2019 (COVID-19) pandemic: increased transmission in the EU/EEA and the UK, Rapid risk assessment—sixth update. https://www.ecdc.europa.eu/en/publications-data/rapid-risk-assessment-novel-coronavirus-disease-2019-covid-19-pandemic-increased. Accessed 12 Mar 2020. 10.2807/1560-7917.ES.2020.25.12.2003261 PMC7118344 32234119

[2] COVID-19 Investigation Team. Clinical and virologic characteristics of the first 12 patients with coronavirus disease 2019 (COVID-19) in the United States. Nat Med. 2020;26(6):861–8. 10.1038/s41591-020-0877-5 PMC12755114 32327757

[3] Zheng S, Fan J, Yu F, Feng B, Lou B, Zou Q (2020). Viral load dynamics and disease severity in patients infected with SARS-CoV-2 in Zhejiang province, China, January–March 2020: retrospective cohort study. BMJ.

[4] Weilie C, Yun L, Xiaozhen Y, Xilong D, Yueping L, Xiaoli C (2020). Detectable 2019-nCoV viral RNA in blood is a strong indicator for the further clinical severity. Emerg Microbes Infect..

[5] Houldsworth A (2017). Exploring the possibility of arthropod transmission of HCV. J Med Virol..

[6] Ling J, Persson Vinnersten T, Hesson JC, Bohlin J, Roligheten E, Holmes EC (2020). Identification of hepatitis C virus in the common bed bug—a potential, but uncommon route for HCV infection?. Emerg Microbes Infect..

[7] Barbazan P, Thitithanyanont A, Missé D, Dubot A, Bosc P, Luangsri N (2008). Detection of H5N1 avian influenza virus from mosquitoes collected in an infected poultry farm in Thailand. Vector Borne Zoonotic Dis..

[8] World Health Organization. Coronavirus disease (COVID-19) advice for the public: Myth busters 2020. https://www.who.int/emergencies/diseases/novel-coronavirus-2019/advice-for-public/myth-busters. Accessed 22 May 2020.

[9] Xia H, Atoni E, Zhao L, Ren N, Huang D, Pei R (2020). SARS-CoV-2 does not replicate in* Aedes* mosquito cells nor present in field-caught mosquitoes from Wuhan. Virol Sin..

[10] Huang YS, Vanlandingham DL, Bilyeu AN, Sharp HM, Hettenbach SM, Higgs S (2020). SARS-CoV-2 failure to infect or replicate in mosquitoes: an extreme challenge. Sci Rep.

[11] Riccardo F, Bolici F, Fafangel M, Jovanovic V, Socan M, Klepac P (2020). West Nile virus in Europe: after action reviews of preparedness and response to the 2018 transmission season in Italy, Slovenia Serbia and Greece. Glob Health.

[12] Chihota CM, Rennie LF, Kitching RP, Mellor PS (2001). Mechanical transmission of lumpy skin disease virus by *Aedes aegypti* (Diptera: Culicidae). Epidemiol Infect..

[13] Otake S, Dee SA, Rossow KD, Moon RD, Pijoan C (2002). Mechanical transmission of porcine reproductive and respiratory syndrome virus by mosquitoes, *Aedes vexans* (Meigen). Can J Vet Res..

[14] Garrett-Jones C, Grab B. The assessment of insecticidal impact on the malaria mosquito's vectorial capacity, from data on the proportion of parous females. Bull World Health Organ. 1964;31(1):71-86. PMC2555159 14230896

[15] Fortuna C, Remoli ME, Di Luca M, Severini F, Toma L, Benedetti E (2015). Experimental studies on comparison of the vector competence of four Italian *Culex pipiens* populations for West Nile virus. Parasites Vectors.

[16] Stefanelli P, Faggioni G, Lo Presti A, Fiore S, Marchi A, Benedetti E, et al. Whole genome and phylogenetic analysis of two SARS-CoV-2 strains isolated in Italy in January and February 2020: additional clues on multiple introductions and further circulation in Europe. Euro Surveill. 2020;25(13):2000305. 10.2807/1560-7917.ES.2020.25.13.2000305. 10.2807/1560-7917.ES.2020.25.13.2000305 PMC7140597 32265007

[17] Centers for Disease Control and Prevention. Novel coronavirus (2019-nCoV) realtime rRT-PCR panel primers and probes. https://www.cdc.gov/coronavirus/2019-ncov/lab/rt-pcr-panel-primer-probes.html. Accessed 6 June 2020.

[18] Corman VM, Landt O, Kaiser M, Molenkamp R, Meijer A, Chu DK (2020). Detection of 2019 novel coronavirus (2019-nCoV) by real-time RT-PCR. Euro Surveill.

[19] Ahn DG, Shin HJ, Kim MH, Lee S, Kim HS, Myoung J (2020). Current status of epidemiology, diagnosis, therapeutics, and vaccines for novel Coronavirus disease 2019 (COVID-19). J Microbiol Biotechnol..

[20] Zhang W, Du RH, Li B, Zheng XS, Yang XL, Hu B (2020). Molecular and serological investigation of 2019-nCoV infected patients: implication of multiple shedding routes. Emerg Microbes Infect..

[21] Clements AN. The biology of mosquitoes volume 1: development, nutrition and reproduction. London: Chapman & Hall; 1992.

[22] Janeh M, Osman D, Kambris Z (2017). Damage-induced cell regeneration in the midgut of* Aedes albopictus *mosquitoes. Sci Rep.

